# Genome-wide association analysis of seedling traits in diverse Sorghum germplasm under thermal stress

**DOI:** 10.1186/s12870-016-0966-2

**Published:** 2017-01-13

**Authors:** Ratan Chopra, Gloria Burow, John J. Burke, Nicholas Gladman, Zhanguo Xin

**Affiliations:** Plant Stress & Germplasm Development Unit, Cropping Systems Research Laboratory, USDA-ARS, Lubbock, TX 79415 USA

**Keywords:** Sorghum association panel, Genome wide association, Thermal stress, Temperature resilience, Anthocyanin, Gene network

## Abstract

**Background:**

Climate variability due to fluctuation in temperature is a worldwide concern that imperils crop production. The need to understand how the germplasm variation in major crops can be utilized to aid in discovering and developing breeding lines that can withstand and adapt to temperature fluctuations is more necessary than ever. Here, we analyzed the genetic variation associated with responses to thermal stresses in a sorghum association panel (SAP) representing major races and working groups to identify single nucleotide polymorphisms (SNPs) that are associated with resilience to temperature stress in a major cereal crop.

**Results:**

The SAP exhibited extensive variation for seedling traits under cold and heat stress. Genome-wide analyses identified 30 SNPs that were strongly associated with traits measured at seedling stage under cold stress and tagged genes that act as regulators of anthocyanin expression and soluble carbohydrate metabolism. Meanwhile, 12 SNPs were significantly associated with seedling traits under heat stress and these SNPs tagged genes that function in sugar metabolism, and ion transport pathways. Evaluation of co-expression networks for genes near the significantly associated SNPs indicated complex gene interactions for cold and heat stresses in sorghum. We focused and validated the expression of four genes in the network of *Sb06g025040*, a basic-helix-loop-helix (*bHLH*) transcription factor that was proposed to be involved in purple color pigmentation of leaf, and observed that genes in this network were upregulated during cold stress in a moderately tolerant line as compared to the more sensitive line.

**Conclusion:**

This study facilitated the tagging of genome regions associated with variation in seedling traits of sorghum under cold and heat stress. These findings show the potential of genotype information for development of temperature resilient sorghum cultivars and further characterization of genes and their networks responsible for adaptation to thermal stresses. Knowledge on the gene networks from this research can be extended to the other cereal crops to better understand the genetic basis of resilience to temperature fluctuations during plant developmental stages.

**Electronic supplementary material:**

The online version of this article (doi:10.1186/s12870-016-0966-2) contains supplementary material, which is available to authorized users.

## Background

Temperature fluctuations during the growing seasons of a crop critically affect plant development and adversely influence the crop production [1]. For example at seedling stage, chilling temperatures slow down growth, generally as a result of reduced enzymatic activities [2, 3], while high temperatures can accelerate growth but with longer exposure has detrimental effects on viability and growth [4]. High temperature stresses induces reactive oxygen species (ROS) production, resulting in oxidative damage and cell death [5]. Impacts of thermal stresses reinforce the need to understand how underlying genetic variation affects the response of major crops to a range of thermal stress, and how genetic diversity can be utilized to develop germplasm with greater fitness against temperature fluctuations.

The introduction and breeding of traits for improved tolerance to abiotic stress is of continuous interest in cereal crops such as sorghum, maize, wheat, and barley for the purposes of sustained production. This has become more necessary in a cereal crop such as sorghum, which is likely cultivated under marginal conditions and thermal stresses. Improving cold stress tolerance in sorghum seedlings will be advantageous for early-season planting and to provide robust stand establishment, whereas heat tolerance can improve the survivability of seedlings during periodic high temperature that occurs in the regular planting season in the US sorghum belt.

Cool ambient air and soil temperatures are major abiotic stressors affecting plant growth and development, and are of great importance in northern latitudes such as USA, Europe and China. Cold stress is known to induce the biosynthesis of flavonoids, anthocyanins, and phenyl-propanoids [6]. Anthocyanin content in leaf was reported to be positively correlated with cold tolerance in some *Arabidopsis* ecotypes [7]. Higher levels of anthocyanin, proposed as the blue light absorbing flavonols in the leaf, ensure that chlorophyll is not over-excited under extreme conditions of cold [8–10], suggesting role of anthocyanin as defense machinery against the cold-induced damage. In sorghum, variation for cold tolerance at genetic level is being evaluated through bi-parental mapping [11, 12] and association mapping approaches [13]. However, the link between cold response and anthocyanin level in sorghum has not been explored to date.

Similarly, heat stress limits the growth and development of sorghum seedling at high temperature (30–40 °C) by further inhibiting photosynthesis [14, 15]. A short period of heat stress is sufficient to provoke severe cellular injury. Chlorophyll synthesis is sensitive to heat stress and is an indicator of heat-stress induced injury. In plants, heat is a major abiotic instigator for the accumulation of ROS, which are detrimental to plant cells causing damage to valuable biomolecules like sugars, lipids, and membranes [16]. Heat tolerant cultivars would be a key improvement for breeding programs to address the adverse effects of abiotic stress on crops [17, 18]. Variation for tolerance to heat stress is observed in sorghum but this remains to be analyzed for use in selection and breeding.

Both cold and heat tolerance are complex quantitative traits, involving multiple regulatory gene mechanisms and metabolic pathways. Therefore, it is important to analyze primary or secondary metabolites along with the morphological and physiological traits such as seedling weight and length that varies with thermal stress conditions. In corn, association studies using seedling traits and chlorophyll phenotypic evaluations were performed in panels to identify QTL and SNP that conditions cold tolerance in seedlings [19]. Further, genetic and molecular basis for cold and heat tolerance traits are necessary to understand the mechanisms and gene regulatory networks underlying tolerance traits at seedling stages in sorghum.

Understanding such complex and coordinated networks requires an establishment of genomic resources for abiotic stress tolerance traits using integrated genomics approach of associating mapping, bi-parental mapping and expression analysis. Whole-transcriptome sequencing can help in constructing putative transcriptional networks; and few studies in sorghum have elucidated differential expression patterns under cold [20], drought [21, 22], and nitrogen stress [23]. Genome-wide gene expression analysis has recently aided in dissecting the impacts of abiotic stress at the molecular level. However, the high abundance of the differentially expressed genes makes it harder to find most critical genes involved in stress defense for a particular genotype. Thus knowledge of the impact of genetic variation on stress response can be improved by combining prior molecular information through genetics (bi-parental/association mapping) in combination with transcriptional networks generated from RNASeq data.

In sorghum, association mapping has been employed to identify association between genome regions or hot spots in the form of single nucleotide polymorphism and different traits, including grain quality [24], plant height [25], stalk rot [26], grain flavonoid pigmentation traits [27], seed size [28], dhurrin content [29], and cold germination [13]. Most of these studies reinforce that association mapping has proved as a powerful approach for dissecting complex traits in sorghum. Therefore a detailed analysis of association between seedling morpho-physiological and metabolite responses to cold and heat stresses, and that of specific genome regions of interest will be highly useful in developing molecular markers for stress-tolerance and is anticipated to enhance the efficiency of traditional breeding programs.

In this study, 300 diverse accessions of sorghum were used to conduct association analysis of seedling phenotypic variation during cold and heat stress treatments with the aim to highlight genetic differences that conditions adaptation to thermal stress. A detailed analysis of the genes present in the QTL regions was carried out to identify candidate genes involved in adaptation to cold and heat stress. Specifically, the SNPs associated with the cold stress traits were validated in a set of genotypes with different tolerance to determine the haplotypes. The expression networks were also evaluated to elucidate putative pathway for thermal stress tolerance.

## Results and discussion

### Phenotypic variation and correlations among the cold and heat response traits

The summary of statistics of the phenotypic data gathered in this study indicated that the different germplasm of sorghum association panel displayed wide range of diversity both under cold and heat stress (Table 1). The heritability values ranged from moderate (0.32 to 0.44) for a number of seedling traits and high for root and anthocyanin under cold stress (Table 1). The heritability for morpho-physiological traits measured under heat stress was also in the moderate range of 0.39–0.53 (Table 1). The analysis of variance showed that genotypes are significantly different for all traits analyzed (Table 1). Genotypes explained the largest proportion of variance among all sources of variation.

**Table 1:** Summary statistics expressed as mean and heritability for traits evaluated for genome wide association studies of the sorghum associaiotn panel under stress at seedling stage. Evaluation of analysis of variance is included in the table to assess the contributionof genotypes

				Analysis of Variance	
Thermal stress	Traits Measured	Mean (±SD)	Heritability	Sums of Squares	Mean Squares
Cold Stress	Shoot weight (mg)	41.00 (±12.00)	0.38	34,068	119.959***
	Root weight (mg)	35.00 (±15.00)	0.32	73,015	257.096***
	Shoot length (cm)	49.48(±10.56)	0.44	28,899	101.760***
	Root length (cm)	105.46(±23.67)	0.61	139,911	492.647***
	Anthocyanin levels (abs at 530 nm/shoot fresh weight – mg)	7.00 (±5.00)	0.60	7,482	26.348***
Heat Stress	Shoot weight (mg)	40.00 (±13.00)	0.47	46,670	164.334***
	Root weight (mg)	32.00 (±13.00)	0.53	50,503	177.828***
	Shoot length (cm)	51.51 (±15.02)	0.47	64,049	225.526***
	Root length (cm)	124.26(±28.59)	0.39	239,073	841.807***
	Chlorophyll A (abs at 663 nm/shoot fresh weight – mg)	32.87(±21.26)	0.52	104,161	384.361***
	Chlorophyll B (abs at 646 nm/shoot fresh weight – mg)	10.72(±6.63)	0.49	9,957	36.742***
	Total Chlorophyll (abs at 470 nm/shoot fresh weight – mg)	43.58(±27.72)	0.52	175,359	647.084***

***- statistically significant -*p*-value of 0.0001

**Fig. 1:**
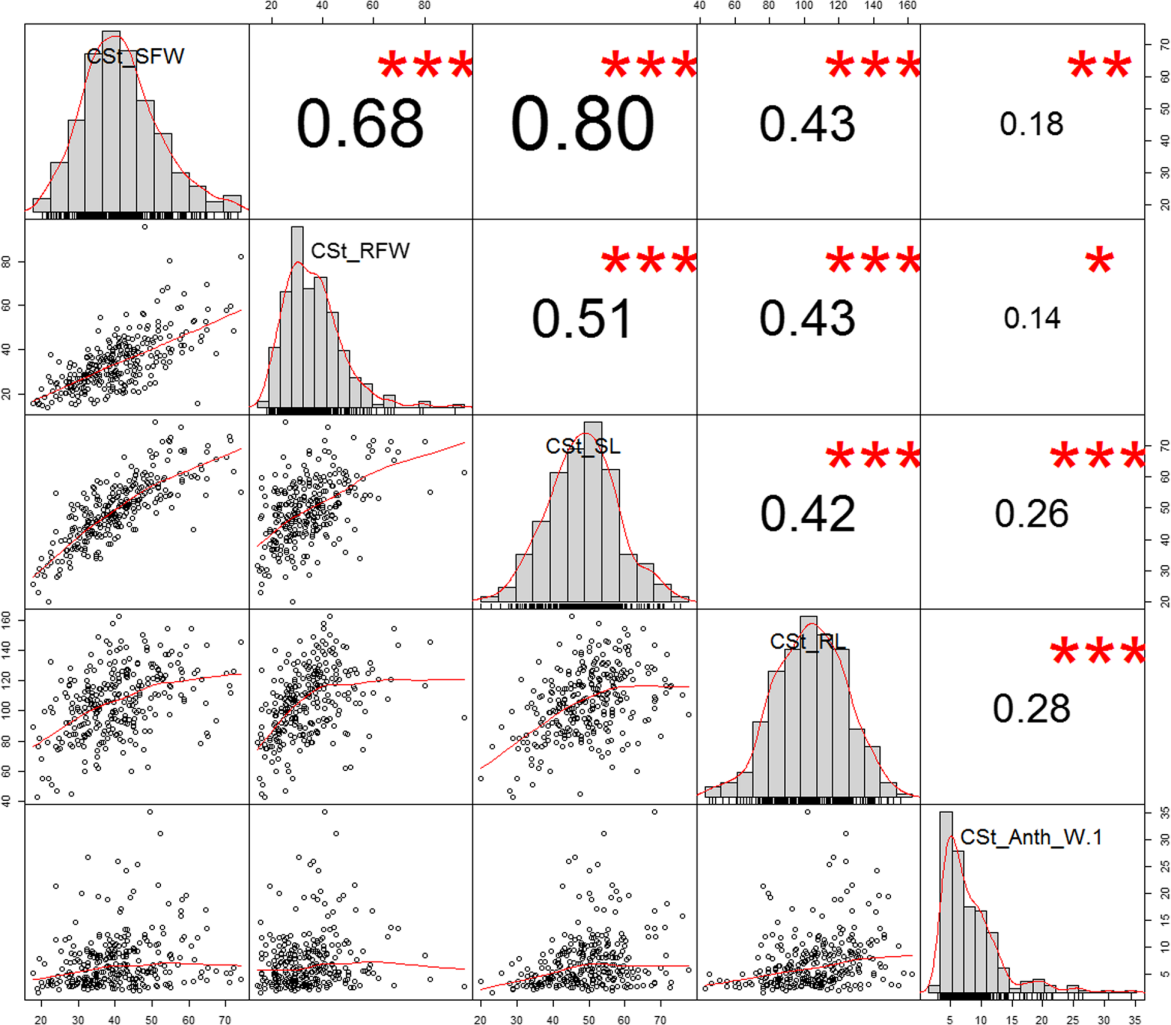
Variation and Pearson pairwise correlations among cold stress related traits. Histograms for shoot weight, root weight, shoot length, root length, and anthocyanin levels are displayed along the diagonal

**Fig. 2:**
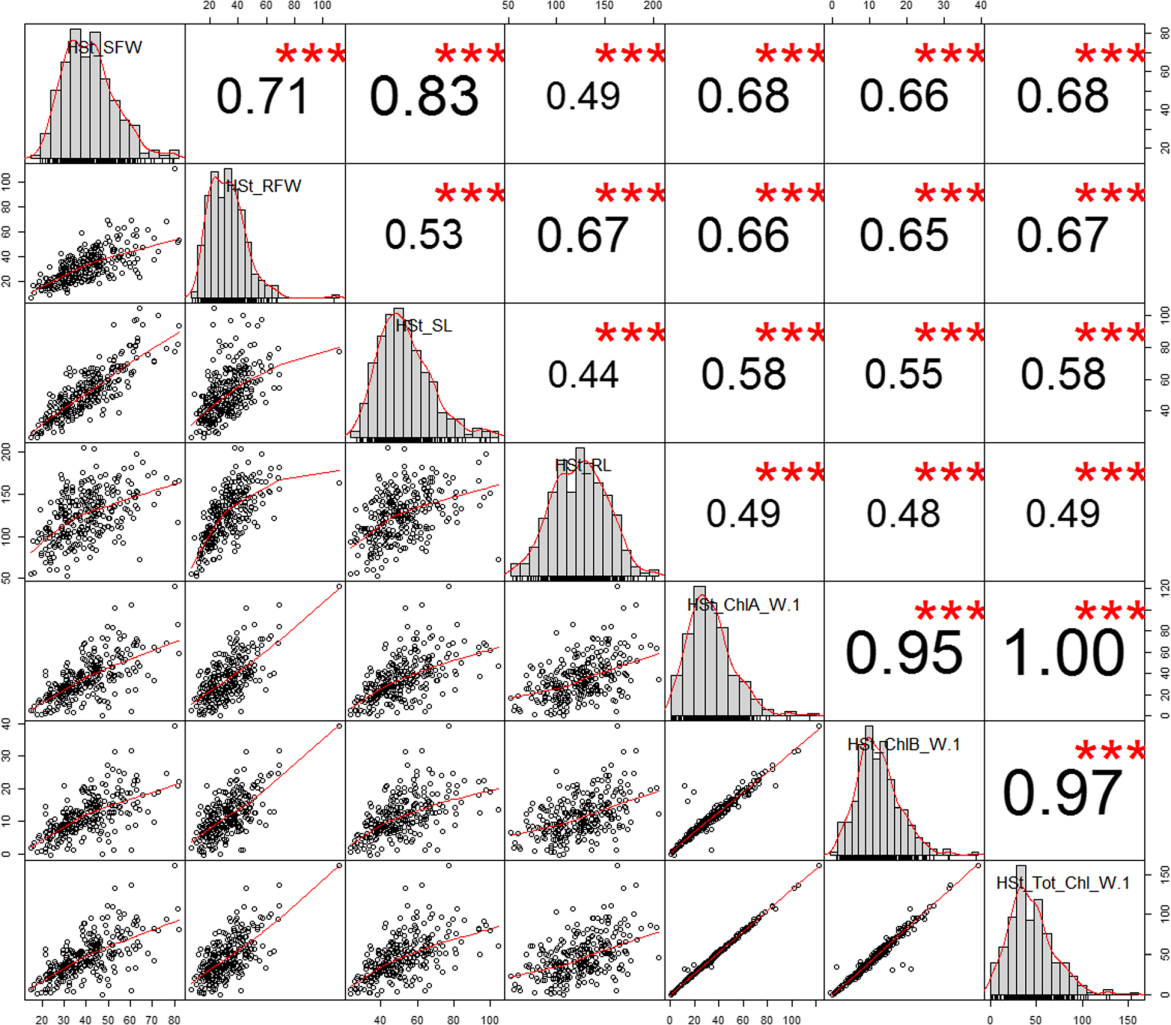
Variation and Pearson pairwise correlations among heat stress related traits. Histograms for shoot weight, root weight, shoot length, root length, chlorophyll A, chlorophyll B, and total chlorophyll are displayed along the diagonal

The level of variation observed for various traits assessed is reflected in the histograms under cold and heat stress (Figs. 1, 2). Notably, under cold stress the correlation between the traits was higher in shoot and root measurements while the correlation of anthocyanin levels with the others traits (including root and shoot features) were moderate and ranged only from 0.142 to 0.305 (Fig. 1). The correlation analysis among the traits under heat stress was also performed (Fig. 2). Seven traits under heat stress treatment had significant correlations, which ranged from 0.451 to 0.997 between each other. However, the comprehensive Pearson correlation analysis of traits between cold and heat stress showed less significant correlations between each other (Additional file 1), indicating that sorghum seedlings exhibited different responses under these two temperature stress conditions at phenotypic or biochemical levels.

These results suggest that the observed genetic variations underlying cold and heat stress responses at the seedling stage are biologically meaningful. Thus, molecular markers associated with the traits could be applicable in sorghum breeding for improving cold and heat tolerance separately.

### Genome-wide association analysis under cold and heat stress

Early-season cold and heat tolerance of sorghum seedlings is a pre-requisite for better crop establishment. Several methods have been used to determine early stage cold tolerance in sorghum [12, 13, 30–32] and Gosavi et al. [19] has assessed heat tolerance of sorghum seedlings for a few genotypes. However, no significant efforts have been made to dissect the genetic mechanisms for heat tolerance of seedlings using large populations or germplasms in sorghum. Broad variability for germination and emergence is a great advantage in breeding sorghum for adaptation [11, 12], therefore we used a SAP to assess the variability among sorghum genotypes and find association for the traits measured under both stresses.

To understand whether there is common allelic variation that could explain differences under thermal stresses, we utilized community resource genotype data for the SAP [33] (www.morrislab.org/data). A total of 265,487 SNPs were utilized for GWAS with the seedling traits data under controlled thermal stress. The phenotypic data for five and seven seedling traits for cold and heat stress respectively, utilized for the analysis are provided in the Additional file 2.

**Fig. 3:**
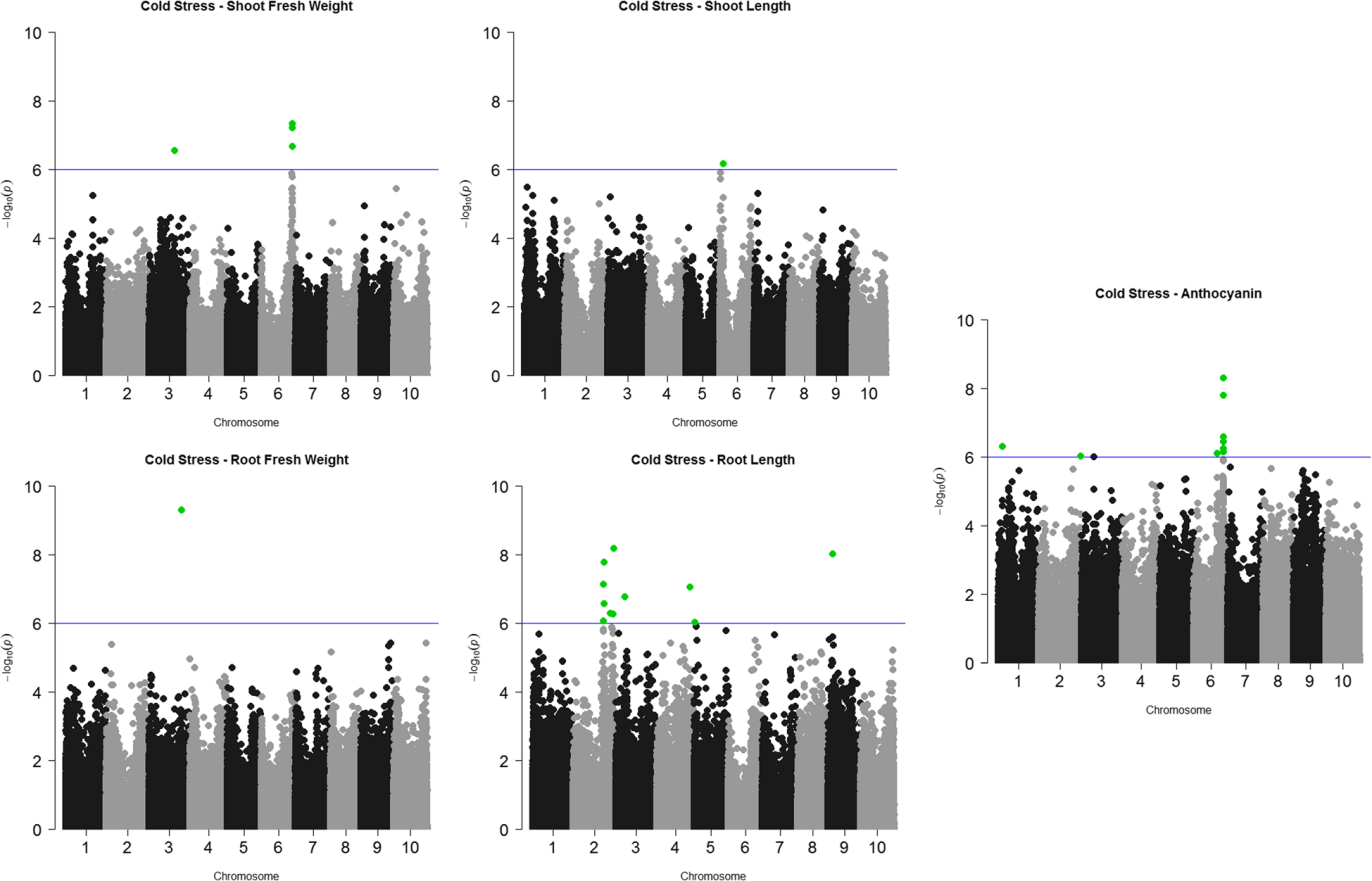
Manhattan plots for significant marker-trait association under cold stress

For cold stress, we evaluated five different traits which included shoot length, shoot weight, root length, root weight, and anthocyanin content followed by association analysis for these traits with 265 K SNPs (Fig. 3). A total of 30 SNPs were significantly associated with the five traits measured with FDR values ≤0.03 (Table 2). Ten SNPs were significantly associated with anthocyanin content of fresh leaf and 13 SNPs were significantly associated with the root length (Table 3). Notably, the significant SNPs that indicate marker-trait associations (MTA) for the anthocyanin content and root length were observed mainly on Chr02 and Chr06 (Fig. 3). Associations for shoot length (5), shoot weight (1) and root weight (1) were found mostly on Chr03 and Chr06 (Fig. 3).

**Table 2:** List of the numbers of significant association for the traits measured under both heat and cold stress including the lowest probability value and false discovery rate (FDR) analysis

Trait (and designation)	Number of significantly associated SNPs*	Lowest *P*-value	Lowest FDR Adjusted values
Cold stress - shoot weight (CSt_SFW)	5	4.69E-08	0.0081
Cold stress - root weight (CSt_RFW)	1	5.11E-10	0.0001
Cold stress - shoot length (CSt _SL)	1	6.99E-07	0.1709
Cold stress - root length (CSt_RL)	13	6.60E-09	0.0012
Cold stress - anthocyanin levels/weight(CSt_Anth_W^−1^)	10	4.89E-09	0.0012
Total	30		
Heat stress - shoot weight (HSt_SFW)	7	2.39E-07	0.0158
Heat stress - root weight (HSt_RFW)	-	-	-
Heat stress - shoot length (HSt_SL)	3	4.56E-07	0.0702
Heat stress - root length (HSt_RL)	-	-	-
Heat stress - Chlorophyll A/fresh weight (HSt_ChlA_W^−1^)	-	-	-
Heat stress - Chlorophyll B/fresh weight (HSt_ChlB_W^−1^)	2	4.50E-07	0.119
Heat stress - Total chlorophyll/fresh weight (HSt_Tot_Chl_W^−1^)	-	-	-
Total	12		

*Total number of significantly associated SNPs with the traits measured during thermal stresses at *p*-value of 1E-06

**Table 3:** List of significant SNP associations, the genes tagged by significant SNPs and information from the genome wide analysis of sorghum germplasm under cold stress at seedling stage

Trait	SNP	FDR Adjusted *p*-Values*	R^2^ Value **	Nucleotide Variation	Distance to nearest gene (bp) ***	Nearest Gene ID
CSt_SFW	S6_57170126	0.008	10.73	C/G	(−)588	*Sb06g028380*
CSt_SFW	S6_57163269	0.008	10.26	A/G	149	*Sb06g028370*
CSt _SFW	S6_57302179	0.015	10.30	T/C	0	*Sb06g028540*
CSt _SFW	S6_57163127	0.015	12.62	A/G	291	*Sb06g028370*
CSt _SFW	S3_47228001	0.015	−17.48	T/C	1973	*Sb03g023720*
CSt _RFW	S3_59699677	0.000	0.06	T/A	930	*Sb03g031320*
CSt _SL	S6_5762763	0.171	8.75	T/A	768	*Sb06g002877*
CSt _RL	S2_73739699	0.001	−20.80	T/A	(−)127	*Sb02g039680*
CSt _RL	S9_8614594	0.001	−14.86	G/A	2098	*Sb09g006020*
CSt _RL	S2_56602226	0.001	−12.79	G/A	543	*Sb02g023140*
CSt _RL	S2_54575799	0.005	11.99	A/G	(+)889	*Sb02g022120*
CSt _RL	S4_60946340	0.005	−25.13	G/C	569	*Sb04g030940*
CSt _RL	S3_16201529	0.008	−16.12	G/C	495	*Sb03g013220*
CSt _RL	S2_56566217	0.010	12.65	C/G	(+)398	*Sb02g023120*
CSt_RL	S2_67807062	0.015	−12.04	C/A	-	*-*
CSt_RL	S2_72583772	0.015	14.48	C/T	(+)2810	*Sb02g038300*
CSt_RL	S2_72833569	0.015	12.10	C/T	(+)2150	*Sb02g038630*
CSt_RL	S2_54593968	0.019	13.13	A/G	(−)156	*Sb02g022140*
CSt_RL	S2_54593969	0.019	13.13	A/G	(−)157	*Sb02g022140*
CSt_RL	S5_1302419	0.019	5.84	C/G	(+)66	*Sb05g001215*
CSt_Anth_W^−1^	S1_8039999	0.022	−42.27	G/T	(−)104	*Sb01g009310*
CSt_Anth_W^−1^	S6_53967668	0.001	−5.71	T/C	77	*Sb06g024943*
CSt_Anth_W^−1^	S6_54057566	0.002	−19.29	G/C	(+)2144	*Sb06g025060*
CSt_Anth_W^−1^	S6_53978420	0.022	−20.33	G/C	661	*Sb06g024960*
CSt_Anth_W^−1^	S6_54030245	0.017	−13.36	G/A	(−)440	*Sb06g025040*
CSt_Anth_W^−1^	S6_53848121	0.023	−5.18	T/C	(+)4385	*Sb06g024820*
CSt_Anth_W^−1^	S6_53791123	0.019	5.21	T/C	(+)2883	*Sb06g024740*
CSt_Anth_W^−1^	S6_43169342	0.023	−47.53	A/G	108	*Sb06g015560*
CSt_Anth_W^−1^	S6_54030254	0.017	13.36	G/T	(−)449	*Sb06g025040*
CSt_Anth_W^−1^	S2_76203986	0.025	36.77	C/T	(−)2259	*Sb02g042450*

*False Discovery Rate (FDR) values for the significantly associated SNP at *p*-value of 1E-06

**Percent trait variation explained by the associated SNPs

***Distance to the nearest annotated gene coordinates in the reference genome

Previous studies in sorghum and other crops have suggested a role for root traits (Bekele et al. [31]; Balota et al. [30]) and anthocyanin levels (Marczak et al. [7]; Hannah et al. [8]) during chilling stress response in sorghum. Recent findings and reports suggested that root establishment could be one of the most important factors for field establishment, next to that of field germination [31]. Furthermore, root biomass under controlled conditions was correlated with the seedling vigor under field conditions [35]. Related studies suggest that root traits could influence the stay-green phenotype and seed yield in sorghum [36]. The significant associations of SNPs for root traits observed in the current study could indicate the possible role of genes tagged by such SNPs in adaptation or tolerance to chilling stress. The establishment of specific SNPs with positive contribution to robust root growth and actual seedling growth under cold temperatures would be beneficial in aiding selection of desirable germplasm with cold stress tolerance during screening with large number of accessions via marker-assisted selection.

**Fig. 4:**
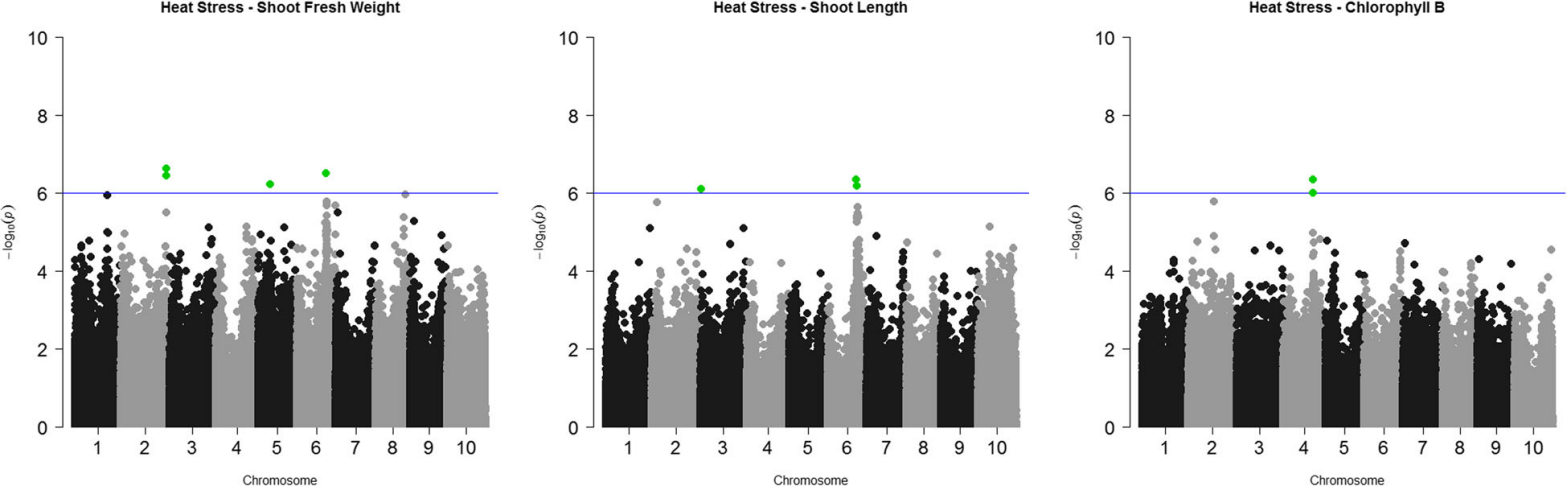
Manhattan plots for significant marker-trait association under heat stress

The heat stress on sorghum seedlings showed association for shoot traits, but none were detected for the root measurements (Table 2). The SNPs identified for shoot fresh weight were present on Chr02, Chr05 and Chr06 with 5 SNPs belonging to the same gene on Chr02 (Fig. 4). Associations for shoot fresh weight explained 10–15% variation in the population and for shoot length from 8 to 25% variation (Table 4). Variation in shoot traits caused by higher temperature suggest that possible favorable alleles in a number of accessions could accelerate the growth, while other haplotypes could have negative effect of retarding plant development under heat stress. It was reported that high temperatures accelerate growth but with longer exposure, viability of seedlings declined [37]. Chlorophyll measurements during heat stress showed significant associations with two SNPs which were present on Chr04, and for shoot length significant SNPs were localized on Chr03 (1) and Chr06 (2) (Fig. 4).

**Table 4:** List of significant SNP associations, the genes tagged by significant SNPs and information from the genome wide analysis of sorghum germplasm under heat stress

Trait	SNP	FDR Adjusted *p*-Values*	*R* ^2^ Value**	Nucleotide Variation	Distance to nearest gene (bp) ***	Nearest Gene ID
HSt_SFW	S2_73084591	0.02	14.71	C/T	(−)900	*Sb02g038910*
HSt_SFW	S2_73084594	0.02	14.60	C/T	(−)903	*Sb02g038910*
HSt_SFW	S2_73084593	0.02	−14.71	G/C	(−)902	*Sb02g038910*
HSt_SFW	S2_73084596	0.02	−14.71	G/A	(−)905	*Sb02g038910*
HSt_SFW	S6_46226536	0.02	−10.48	G/A	(+)847	*Sb06g017060*
HSt_SFW	S2_73084592	0.02	14.60	A/G	(−)901	*Sb02g038910*
HSt_SFW	S5_19670409	0.02	−15.00	G/A	-	*Sb05g009740*
HSt_SL	S6_45982131	0.07	8.69	A/G	(−)318	*Sb06g016880*
HSt_SL	S6_46194160	0.07	−7.96	G/A	(−)127	*Sb06g017025*
HSt_SL	S3_1638378	0.07	−24.38	T/G	302	*Sb03g001820*
HSt_ChlB_W^−1^	S4_48000182	0.12	20.89	G/A	-	*Sb04g020520*
HSt_ChlB_W^−1^	S4_48000349	0.13	20.39	G/A	-	*Sb04g020520*

*False Discovery Rate (FDR) values for the significantly associated SNP at *p*-value of 1E-06

**Percent trait variation explained by the associated SNPs

***Distance to the nearest annotated gene coordinates in the reference genome

The total chlorophyll content measured under heat stress provided association with SNP S4_48000182 and S4_48000349 explaining 20% variance. Associations with the chlorophyll content under heat stress were less significant as compared to the other traits. It is generally accepted that chlorophyll biosynthesis is highly sensitive to heat stress. To confer tolerance to heat-stress induced injury variations among genes involved in the biosynthetic pathway may provide protective mechanisms.

Notably, SNP associated with a number of seedling traits during thermal stress were linked to the variations in sorghum Chr06. For example, anthocyanin content trait (CSt_Anth_W^−1^) during cold stress had eight SNP markers localized on Chr06, and shoot seedling traits under heat and cold stresses also, had eight SNPs localized on Chr06 (Table 3 and 4). A number of significant regions in Chr06 has previously been implicated in other stress tolerance such as ergot resistance [38], drought tolerance [36], sugar metabolism [39], and contrasting photoperiod conditions [40]. These results indicate an important role of genes in Chr06 in sorghum growth and development under various environmental conditions making it an interesting focus for studies of selection.

### Genome scans for candidate genes near significant SNPs

Majority of the SNPs associated with the traits subjected to thermal stresses were either within the gene or a few thousand nucleotides apart. Notably, the resolution of such strong linkage to trait-responsible genes could be possible in our GWAS study because of distinct quantitative measures of response to thermal stress as compared to indirect measures in other studies. Thirty-three putative genes near the 43 associated loci were identified and ﻿are presented with gene annotations including possible functions, in Table 5.

**Table 5:** List of candidate genes identified in the study based on proximity to the significant markers identified through GWAS analysis and their description or function obtained from gramene(ww.gramene.org) and phytozome (www.phytozome.net) databases

Gene ID	Annotation
Sb01g009310	methyltransferases
Sb02g022120	HAESA-like 1
Sb02g023140	Cupredoxin superfamily protein
Sb02g023120	P-loop containing nucleoside triphosphate hydrolases superfamily protein
Sb02g022140	Unknown
Sb02g038300	Saccharopine dehydrogenase
Sb02g038630	Unknown
Sb02g039680	alpha/beta-Hydrolases superfamily protein
Sb02g042450	Pentatricopeptide repeat (PPR) superfamily protein
Sb03g013220	Peroxidase superfamily protein
Sb03g023720	Expressed Protein
Sb03g031320	splicing factor-related
Sb04g030940	LisH/CRA/RING-U-box domains-containing protein
Sb05g001215	myb domain protein 61
Sb06g002880	electron transfer flavor protein beta
Sb06g015560	weakly similar to H0717B12.8 protein
Sb06g024740	Nucleotide-diphospho-sugar transferases superfamily protein
Sb06g024820	GRAS family transcription factor
Sb06g024943	expressed protein
Sb06g024960	UDP-Glycosyltransferase superfamily protein
Sb06g025040	basic helix-loop-helix (bHLH) DNA-binding superfamily protein
Sb06g025060	basic helix-loop-helix (bHLH) DNA-binding superfamily protein
Sb06g028370	AICARFT/IMPCHase bienzyme family protein
Sb06g028380	K+ uptake permease 10
Sb06g028540	similar to Putative uncharacterized protein
Sb09g006020	Actin-like ATPase superfamily protein
Sb04g020520	exocyst subunit exo70 family protein F1
Sb02g038910	Pectin lyase-like superfamily protein
Sb06g017060	homeobox protein 22
Sb05g009740	expressed protein
Sb06g016880	magnesium transporter 4
Sb06g017025	Protein of unknown function (DUF668)
Sb03g001820	GDSL-like Lipase/Acylhydrolase superfamily protein

For cold stress conditions, we found that some genes were annotated as transcription factors and others belong to metabolic biosynthesis or transport. Two *bHLH* transcription factors, *Sb06g025040* and *Sb06g0254060* belong to an anthocyanin regulatory mechanism and were associated with the anthocyanin levels measured under cold conditions. Transcription factors such as *bHLH* are suggested to be involved in stress-adaptive mechanisms; recent studies have shown the role of such transcription factors at least in part to cold tolerance, by positively regulating ROS removal [41–43]. Similarly, *Sb03g031320,* a C2H2 zinc finger protein with a putative splicing function, is associated with root fresh weight under cold conditions. Zinc finger proteins are also involved in regulating tolerance mechanisms under oxidative stresses [44], and more recently a study showed increases in fresh weight and root length upon cold stress compared to wild-type with the transformation of C2H2 domain gene in *Arabidopsis* [45]. Other transcription factors such as *Sb06g024820* and *Sb05g001215* were also associated with anthocyanin level and root length, respectively. These genes along with the regulatory transcription factors could be involved in membrane stability and tolerance to cold stress.

One of the significant QTLs on Chr06 (S6_54057566) co-localizes with a gene identified in a previous study for coleoptile color [27], which was a few hundred thousand nucleotides apart from the *bHLH* gene (*Sb06g025040*) as in the report of Morris et al. [36]. In the current study, distinct SNPs found in *Sb06g025040* (−440 and −449) associated with quantitative measures of anthocyanin could be placed upstream of the putative *bHLH* regulatory gene. This variant could potentially be present in the cis-regulatory regions of the transcription factor *Sb06g025040* and could be involved in the regulation of anthocyanin levels for maintaining seedling vigor during stress conditions.

For associated variation under heat stress conditions, we found candidate genes with possible functions as membrane protein (*Sb04g020520*), in sugar metabolism (*Sb02g038910*), as transcription factor (*Sb06g017060*), as proteasome (*Sb05g009740*), as ion transporter (*Sb06g016880*), and in lipid metabolism (*Sb03g001820*). The shoot fresh weight was associated with sugar metabolism, proteasome, and ion transporter genes. K^+^ ion transporter genes are involved in programmed cell death and metabolic adjustments [46], whereas proteasomes are known to affect the heat sensitivity in *Arabidopsis* [47].

In this study, although the genomic analysis showed no overlap between associated regions, the genes involved in tolerance to thermal stress were related. Many of the associations were found close to genes related to membrane transport and sugar metabolism, all of which have potential roles in different stress responses. Therefore, many of the associations observed in our study are suggestive of a coordinated response in sorghum seedling during thermal stress. Previous reports in research have suggested that response to cold and heat stresses are coordinated processes between primary and secondary metabolites and transporters [48–50].

### Validation of loci in a germplasm

The validation of significant SNPs was performed in nine sorghum lines to develop markers for potential use in breeding for thermal stress tolerance. Here, we focused on loci associated with seedling cold tolerance traits in a set of breeding lines with their varying tolerance/sensitivity to cold stress. Towards this end, allele-specific KASP primers were designed for each SNP and used for germplasm genotyping. The results showed that nine of 29 associated SNPs consistently ﻿differentiate at least two susceptible lines from the tolerant lines (Additional file 3). These nine variants could be the putative haplotype present in the sorghum that conditions tolerance cold response. These haplotypes were associated with three different traits (anthocyanin levels, shoot and root fresh weights) measured under cold stress. However, further studies are needed to establish any causal relation of these markers to actual tolerance or susceptibility response.

### Co-expression modules and expression patterns

The genes associated with the measured traits in this study could serve as a basis for exploring the thermal stress responsive mechanisms, and therefore it is important to evaluate the regulation and expression of the their gene networks. As it is difficult to evaluate all traits or metabolites affected by stress, expression networks can provide a logical approach towards identifying genes which otherwise could not be detected in GWAS analysis due to limitations in markers density or panel size. In this study the co-expression networks for each of the genes associated with the traits measured under stress were retrieved from http://sorghum.riken.jp/morokoshi/ [51] to decipher biochemical pathways or gene regulatory networks related to significant SNPs (Additional file 4).

We selected a few genes and their networks to understand their role in cold stress. *Sb06g015560* and *Sb06g028370* are involved in amino acid biosynthesis based on their gene networks whereas *Sb03g031320* is functionally associated with spliceosome. The gene *Sb06g024960* is a UDP-Glycosyltransferase superfamily protein involved in zeatin biosynthesis and was negatively associated with the anthocyanin levels. Zeatin biosynthesis genes have been previously reported to be involved in defense mechanism upon cold stress [52, 53]. Cis-zeatin-type cytokinins are known to regulate the plant growth and the responses to environmental changes [54].

Similarly, S5_19670409 associates with the gene *Sb05g009740* and has a negative effect on fresh shoot weight upon heat stress. *Sb05g009740* has no assigned function but the genes in its network belonged to the proteasome and RNA transport. It is reported that in *Arabidopsis*, proteasome subunit mutants limit 26S proteasome capacity within the cell and can cause heat shock hypersensitivity, and reduced cell division rates [47]. This suggests that *Sb05g009740* and its network of genes are associated with the fresh shoot weight during heat stress.

**Fig. 5:**
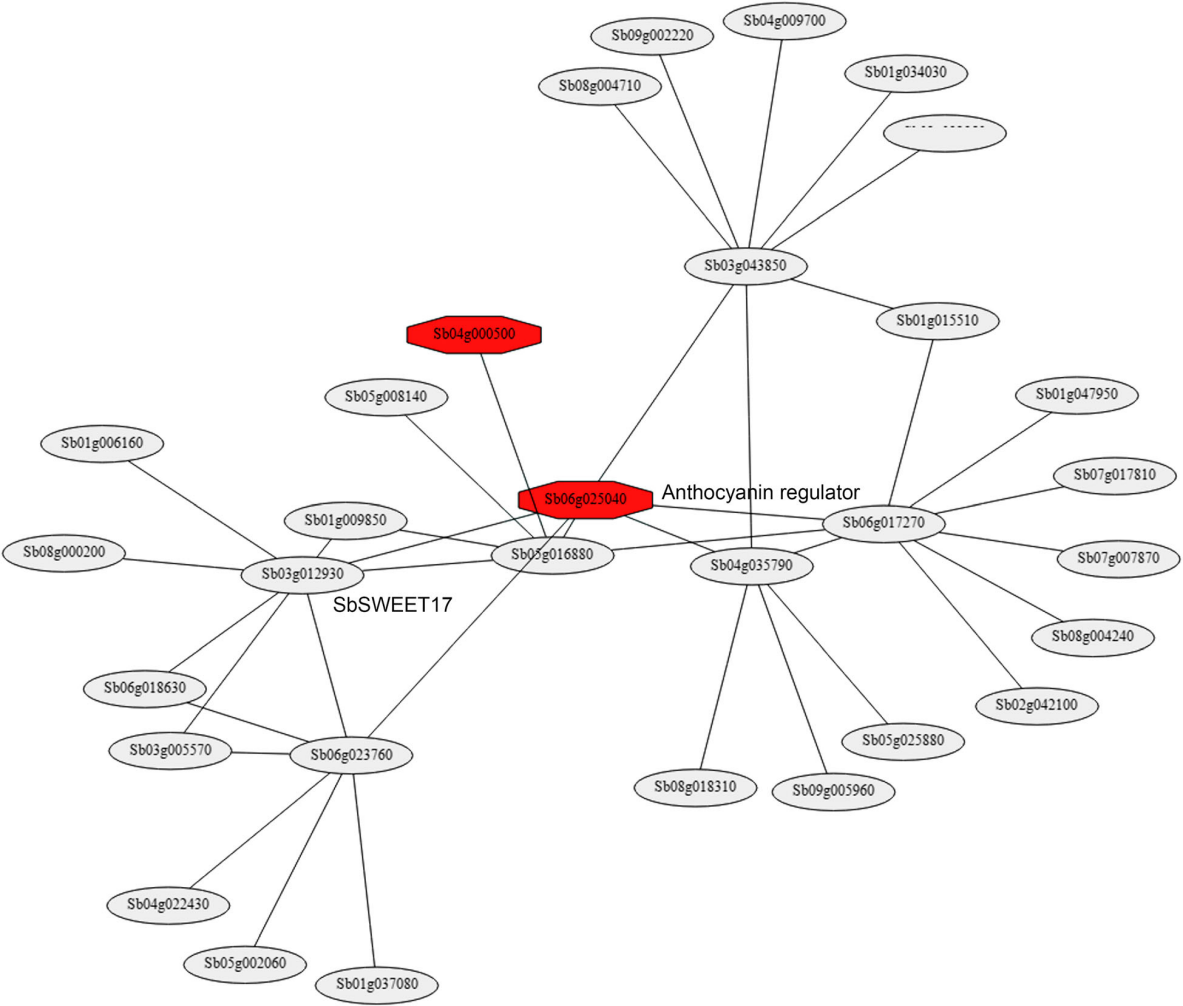
Co-expression module for *Sb06g025040*, one of the associated genes with anthocyanin level under cold stress in sorghum association panel

We also evaluated co-expression network of *Sb06g025040* (*bHLH*) and found that the genes in the network were involved in sugar biosynthesis and transport (Fig. 5). It has been shown that in higher plants, the anthocyanin pathway is regulated by a suite of transcription factors including MYB, *bHLH*, and WD repeat proteins [55–57]. Studies have also reported that the induction of anthocyanin and flavonoid biosynthesis can promote accumulation of metabolites such as sugars and hormones [58, 59].

Based on these networks of the genes, we likewise determined if any differences were found in the expression of the member genes in previous abiotic stress studies in sorghum. To do this, we examined the normalized FPKM values for the associated genes from a RNASeq experiment by Chopra et al. [23] and found that six of the 32 genes showed differential response upon cold stress between the susceptible and tolerant genotypes (Additional file 5). The SNPs associated with the differentially expressed genes *Sb06g025040*, *Sb06g015560*, *Sb06g028370*, and *Sb03g031320* were present within or in very close proximity to the member gene itself (Tables 3 and 4). More importantly, we were able to confirm nucleotide differences between the cold tolerant and susceptible genotypes of sorghum.

**Fig. 6:**
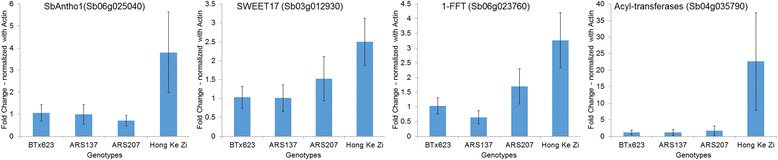
Quantitative-PCR analysis of genes in co-expression network of *Sb06g025040* between cold tolerant (Hong Ke Zi), susceptible line (BTx623), EMS mutants ARS137 and ARS207

Since the differences of haplotype and expression levels were significant for four of the associated genes, we proceeded to to validate their expression levels. We selected *Sb06g025040* transcription factor to validate the expression as the mutation linked to the gene could be in cis-regulatory region. The expression analysis confirmed that gene *Sb06g025040* was relatively upregulated in the tolerant genotype (Fig. 6) upon cold stress. However, the specific role of the gene in cold tolerance will require further molecular characterization. To evaluate if other genes involved in the network of *Sb06g025040* were also affected by cold stress, we selected three other genes (*Sb03g012390*, *Sb06g023760*, and *Sb04g035790*) that were affiliated with *Sb06g025040*. Similar expression patterns were observed between the tolerant and susceptible lines during cold stress (Fig. 6), suggesting a possible role of these genes in cold tolerance of sorghum seedlings. From these results we can propose that *Sb06g025040* and genes within its network are strongly associated with cold tolerance mechanisms. Similarly, other genes such as *Sb06g015560*, *Sb06g028370*, and *Sb03g031320* and their networks can be evaluated to determine their role in cold tolerance.

 We also utilized existing ethyl methyl sulfonate (EMS)- mutants to evaluate effects of induced variation in the associated genes identified. Fortuitously, EMS-induced mutants for *Sb06g025040* and *Sb03g012930* genes from the above network are available from sorghum mutant library [60] for evaluation. Briefly, the ARS207 mutant line had non-synonymous mutation in *Sb06g025040*, and the ARS137 mutant had a non-synonymous mutation in *Sb03g012930* [60]. We performed expression analysis on these mutants to determine their effect on gene expression. On performing qRT-PCR expression analysis on the mutants compared to wild-type, we found down-regulation of *Sb06g025040* in ARS207, but no differences in qRT-PCR data was observed in the ARS137 for *Sb03g012930* (Fig. 6). However, further experiments are required to characterize and understand the functional aspects of these differential gene expression patterns.

## Conclusions

A combined investigation involving seedling traits and metabolite phenotyping, aided by genomics based platforms, identified a number of key associations and their complex network of genes underlying the response of sorghum seedlings to cold and heat stresses. Many of the associated SNPs with seedling traits were within or in very close proximity to stress responsive genes. The evaluation of haplotype differences in selected tolerant and susceptible genotypes for select SNP markers associated with trait variation under cold stress can serve as a possible tool for marker assisted breeding in sorghum. Differential gene expression during thermal stress for the candidate genes tagged aided in assessing whether the haplotypes identified here would be useful in understanding their role for molecular characterization. Finally, we suggest that the application of expression networks along with RNAseq and GWAS analysis, as highlighted in this study, provides a strong approach to elucidate molecular mechanism involved in thermal and other abiotic stress responses in cereal crops.

## Methods

### Plant materials and genotype data

A total of 300 sorghum accessions from the U.S. sorghum association panel- previously described [61], were used in the study (available through the active sorghum germplasm collection at www.ars-grin.gov). The panel is composed of 198 sorghum conversion lines, 46 elite inbreds, and 56 historical accessions of sorghum. The lines are diverse and also represents the major races (kafir, durra, caudatum and bicolor) and working groups of sorghum.

We utilized the community genotype data to perform genome-wide association analysis (www.morrislab.org/data). A total of 265,487 SNPs based on genotype-by-sequencing (GBS) analyses that corresponds to variation in 27,412 annotated genes were utilized [33]. Previous studies demonstrated that this panel and genotype data has sufficient power to dissect complex traits using such populations, including grain yield [62], plant architecture [63] and seed micronutrient composition [64] among other traits.

### Growth conditions and phenotypic evaluation

Evaluation of traits was performed under controlled conditions, where accessions were planted in ragdoll set up in a Mason jar filled with 300 ml of deionized water at constant 14 °C for chilling stress using controlled chamber cabinets (Percival growth cabinets). Each entry was represented by four replicates, and each replicate consist of five seedlings in a mason jar. A completely randomized design was used to set up the experiment. The seedlings were allowed to grow under 24 h light conditions for 7 days. The experiments were conducted in batches of 100 entries to facilitate handling of the large number of accessions in SAP. The following traits were evaluated for the entries of the association panel under chilling stress; shoot fresh weight (SFW) and root fresh weight (RFW), shoot length (SL) and root length (RL) and anthocyanin level (Anth_W^−1^). Anthocyanin was extracted from the finely ground shoot of 3 pooled seedlings using 1.0 mL of acidic methanol (80% methanol, 0.01 M HCL) for 24 h in the dark. Anthocyanin content was estimated based on absorbance of methanolic extract at 530 nm. Levels of anthocyanin were expressed as absorbance at 530 nm per mg fresh weight.

Heat Stress was evaluated at 37 °C in the Percival growth cabinets for 7 days. Each entry was represented by four replicates, and each replicate consist of five seedlings in a mason jar. A completely randomized design was used to set up the experiment. The seedlings were allowed to grow under 12/12 h light–dark conditions for 7 days. Shoot fresh weight (SFW), root fresh weight (RFW), shoot length (SL), and root length (RL) were measured at 7 days after planting. Chlorophyll was extracted from the fresh shoot of three pooled seedlings using 80% acetone and centrifuged at 10,00 rpm. Absorbance of resulting supernatant was determined at 470 nm, 646 nm and 663 nm for each entry.

### Association analyses

Genome-wide association analysis was performed using CMLM in combination with SUPER model to determine marker-trait associations. The compressed mixed linear model (CMLM) involves genetic marker–based kinship matrix modeling of random effects used jointly with population structure estimated by principal components analysis (PCA) to model fixed effects [34, 65, 66]. SUPER model [67] extracts a small subset of SNPs from CMLM and uses them in FaST-LMM method. This method increases statistical power and is computationally advantageous. The compression level and optimal number of principal components that adequately explain population structure were previously determined by the Genomic Association and Prediction Integrated Tool [34]. Log quantile—quantile (QQ) *p*-value plots for 265,487 single SNP tests of association implied that there were few systematic sources of spurious association using CMLM with SUPER model. As several studies have suggested varying number of LD blocks in sorghum, LD range of 3–30 kb was used for the analysis.

### DNA isolation, primer design and genotyping of SNPs

To validate bi-allelic SNPs in a total of nine sorghum genotypes, genomic DNA was isolated from seed samples using Qaigen miniprep plant kit. About 150 bp upstream and downstream of the SNP loci were extracted from the available genome sequence for use in primer design. These sequences were passed through primer3 program with the option of allele-specific and flanking primer method and the size range of 60–100 nucleotides (http://probes.pw.usda.gov/ cgi-bin/batchprimer3/batchprimer3.cgi). Primer sequences are provided as an Additional file 6. End point genotyping using KASP was performed as previously described in Chopra et al. [23].

### Genes in QTL regions and Co-expression network

Associated SNPs were scanned for the nearby genes in the genomic coordinates using gramene biomart (www.gramene.org) and phytozome v.10 (www.phytozome.net). Closest genes in the upstream or downstream of 20 kb were selected and the respective annotations were obtained for each of the genes. Co-expression networks for each of the genes associated was retrieved using the expression database developed by Makita et al. [54]. Top twenty genes in the network for each of the associations have been attached as an Additional file 3.

### Expression analysis using qRT-PCR

For this study 20 seeds for each of the cultivar Hong Ke Zi (cold tolerant) and BTx623 (cold sensitive) were planted in ragdoll set up under cold stress temperatures. The temperatures were continuous 14 °C for cold stress using Conviron walk-in growth cabinets. The seedlings were allowed to grow under 14 h light/10 h dark conditions. Leaf tissue samples for RNA extraction were obtained from five seedlings per replicate for Hong Ke Zi and BTx623. Three replicates were extracted separately using the TRIzol reagent and company recommended protocols (Life Technologies, Grand Island, NY). The total RNA samples from each replicate were quantified using Nano Drop and subsequently purified using the RNAeasy mini clean up kit (Qiagen, Valencia, CA). To evaluate the expression differences observed, qRT-PCR primers were designed for four selected genes in the expression module using primer3 (Additional file 7) and sorghum actin gene was used for normalization. Briefly, qRT-PCR was performed using copy-DNA libraries generated from the total RNA using Invitrogen cDNA synthesis kit (Invitrogen, Grand Island, NY). qRT-PCR was performed on the cDNA of the four samples with 3 biological and 3 technical replicates using SybrGreen on LightCycler 480.

## Additional files

Additional file 1: Pearson’s correlation among traits measured with thermal stress application at seedling stage. (XLSX 10 kb)
Additional file 2: Phenotypic measurements of seedling upon heat and cold stress on 300 sorghum association panel. (XLSX 50 kb)
Additional file 3: Validation of SNPs in nine sorghum lines to determine the haplotypes for cold tolerance. (XLSX 10 kb)
Additional file 4: List of top twenty genes in the network of associated genes derived from sorghum gene network porvided by Makita et al. [51]. (XLSX 40 kb)
Additional file 5: List of related RNASeq normalized FPKM values for susceptible (BTx623) and tolerant (Hong Ke Zi) lines during cold stress retrieved from Chopra et al. [20]. (XLSX 10 kb)
Additional file 6: Lis of KASP primers sequences designed for the significant SNPs associated with varaition of seedling traits under thermal stress. (XLSX 10 kb)
Additional file 7: List of qRT-PCR primers used in expression analysis of the selected genes in this study. (XLSX 9 kb)

## Abbreviations

AOV: Analysis of variance
bHLH: Basic-helix-loop-helix (bHLH)
CMLM: Compressed mixed linear model
FDR: False discovery rate
GWAS: Genome-wide association studies
kb: Kilobase of nucleotides
SAP: Sorghum association panel
SNP: Single nucleotide polymorphism
